# Supplementary material for: Matrix stiffness-induced IKBKE and MAPK8 signaling drives a phenotypic switch from DCIS to invasive breast cancer
Source: Cell Commun Signal. 2025 Jun 4;23:269. doi: 10.1186/s12964-025-02276-y (PMC12139146; doi:10.1186/s12964-025-02276-y)
Supplement: Supplementary file 5 — Supplementary Material 5 [file 12964_2025_2276_MOESM5_ESM.docx]

**Supplementary Fig. 1**

**
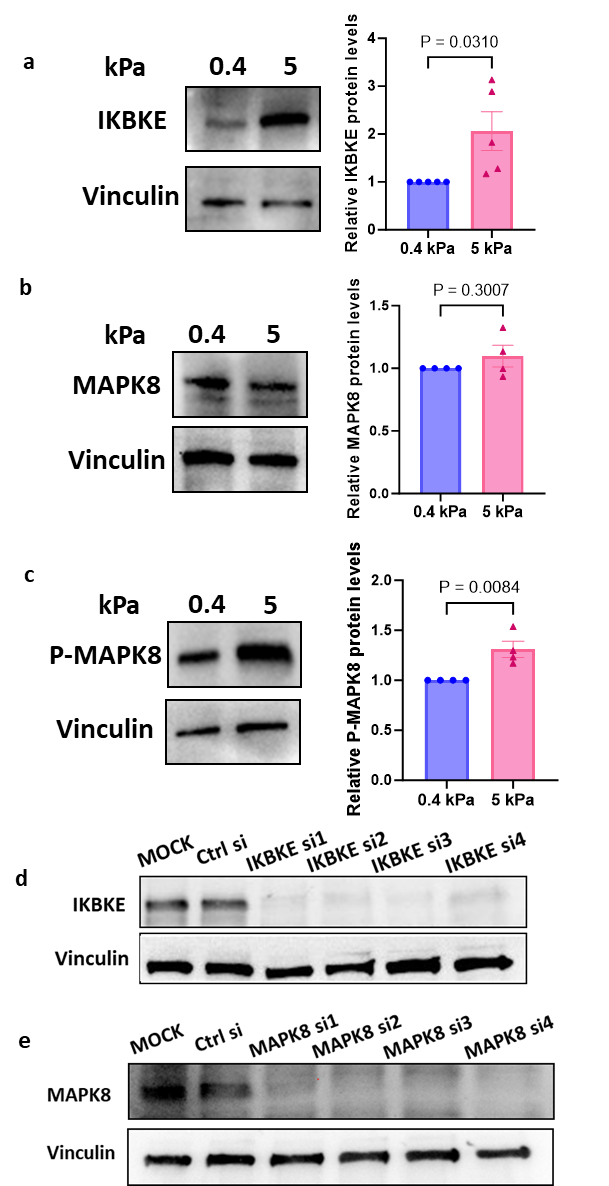
**

**a, b, c,** Immunoblot analysis of IKBKE (a), MAPK8 (b), and phospho-MAPK8 (c) in HCC1143 human breast cancer cells cultured on 0.4 kPa or 5 kPa hydrogels, respectively (left). Densitometric analysis showed target protein levels normalized to loading control (Vinculin) and expressed relative to the low stiffness level (right). Data are presented as mean ± S.E.M., with p-values according to an unpaired t-test. **d, e,** Immunoblot analysis of IKBKE (d) and MAPK8 (e) in HCC1143 cells treated with IKBKE or MAPK8 individual siRNAs, or control siRNA, with Vinculin used as a loading control.

**Supplementary Fig. 2**

**
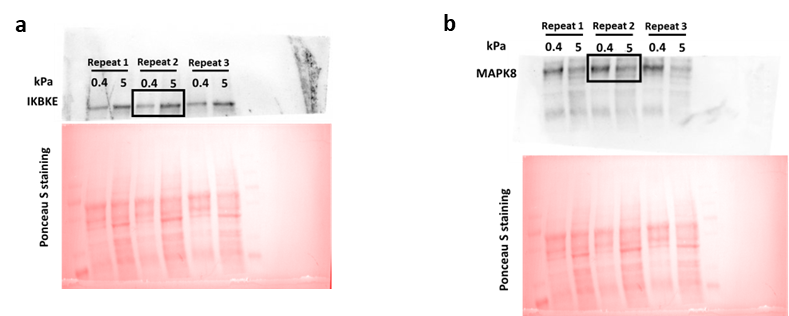
**

**a,** Whole blots and Ponceau S staining for Figure 4a. **b,** Whole blots and Ponceau S staining for Figure 5a left. The membranes were cut horizontally before antibody incubation.


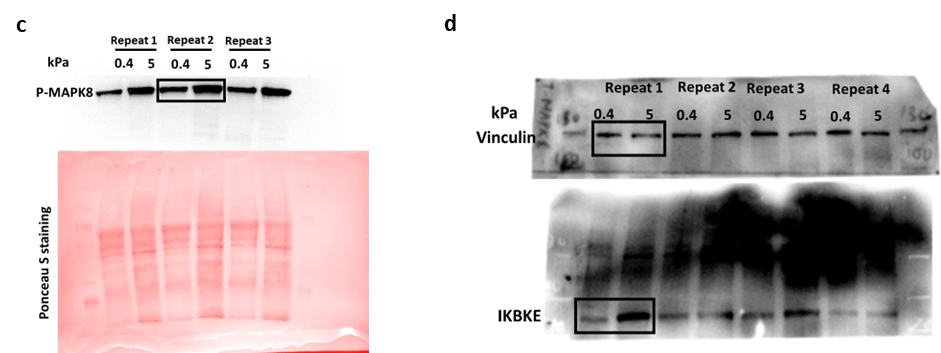


**c,** Whole blots and Ponceau S staining for Figure 5a right. **d,** Whole blots for Supplementary Figure 1a. The membranes were cut horizontally before antibody incubation.


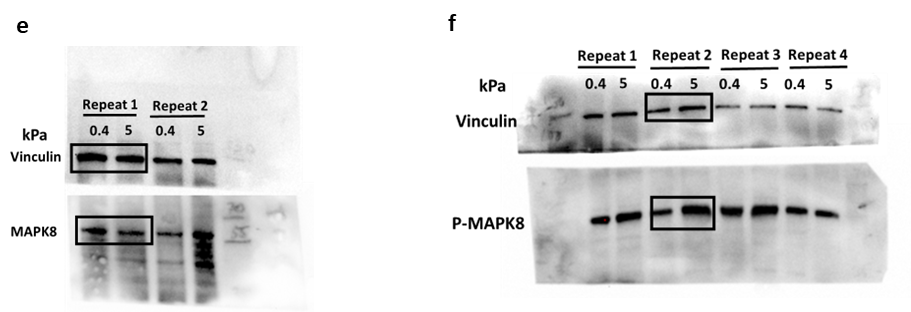


**e,** Whole blots for Supplementary Figure 1b. **f,** Whole blots for Supplementary Figure 1c. The membranes were cut horizontally before antibody incubation.


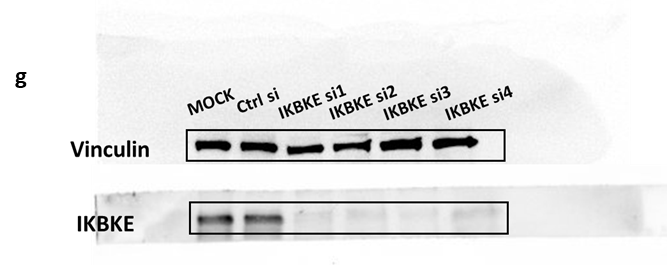


**g,** Whole blots for Supplementary Figure 1d. The membranes were cut horizontally before antibody incubation.


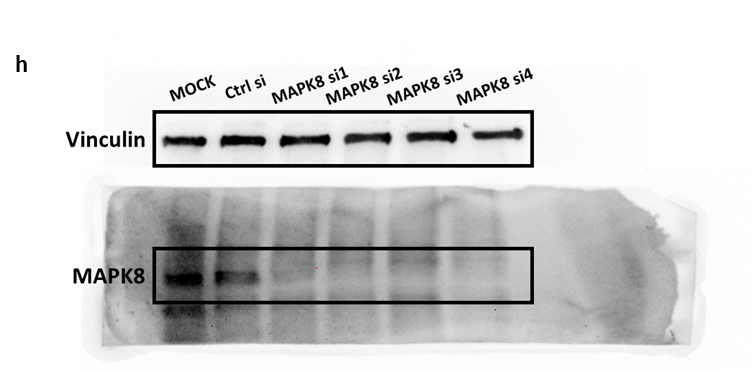


**h,** Whole blots for Supplementary Figure 1e. The membranes were cut horizontally before antibody incubation.
